# Molecular diversity and selective sweeps in maize inbred lines adapted to African highlands

**DOI:** 10.1038/s41598-019-49861-z

**Published:** 2019-09-17

**Authors:** Dagne Wegary, Adefris Teklewold, Boddupalli M. Prasanna, Berhanu T. Ertiro, Nikolaos Alachiotis, Demewez Negera, Geremew Awas, Demissew Abakemal, Veronica Ogugo, Manje Gowda, Kassa Semagn

**Affiliations:** 1 International Maize and Wheat Improvement Center (CIMMYT) - Ethiopia Office, ILRI Campus, CMC Road, Gurd Sholla, P.O. Box 5689, Addis Ababa, Ethiopia; 2 International Maize and Wheat Improvement Center (CIMMYT), ICRAF House, United Nations Avenue, Gigiri, P.O. Box 1041-00621, Nairobi, Kenya; 3 Bako National Maize Research Center, Ethiopian Institute of Agricultural Research (EIAR), Addis Ababa, Ethiopia; 4 Institute of Computer Science, Foundation for Research and Technology-Hellas, Nikolaou Plastira 100, 70013 Heraklion, Crete Greece; 5 Ambo Agricultural Research Center, P.O. Box 37, West Shoa, Ambo Ethiopia; 6 Africa Rice Center (AfricaRice), M’bé Research Station, 01 B.P. 2551, Bouaké 01, Côte d’Ivoire

**Keywords:** Genetic variation, Plant breeding, Plant stress responses

## Abstract

Little is known on maize germplasm adapted to the African highland agro-ecologies. In this study, we analyzed high-density genotyping by sequencing (GBS) data of 298 African highland adapted maize inbred lines to (i) assess the extent of genetic purity, genetic relatedness, and population structure, and (ii) identify genomic regions that have undergone selection (selective sweeps) in response to adaptation to highland environments. Nearly 91% of the pairs of inbred lines differed by 30–36% of the scored alleles, but only 32% of the pairs of the inbred lines had relative kinship coefficient <0.050, which suggests the presence of substantial redundancy in allelic composition that may be due to repeated use of fewer genetic backgrounds (source germplasm) during line development. Results from different genetic relatedness and population structure analyses revealed three different groups, which generally agrees with pedigree information and breeding history, but less so by heterotic groups and endosperm modification. We identified 944 single nucleotide polymorphic (SNP) markers that fell within 22 selective sweeps that harbored 265 protein-coding candidate genes of which some of the candidate genes had known functions. Details of the candidate genes with known functions and differences in nucleotide diversity among groups predicted based on multivariate methods have been discussed.

## Introduction

Maize (*Zea mays* ssp. *mays* L.) is one of the top three crops globally in total production and is cultivated as a multi-purpose crop for food, feed, biofuel, and raw material for synthesis of various industrial products^1^. In Africa, maize is produced on a total area of nearly 37 million hectares, which is about 20% of the total maize area of the world. However, the total maize production for the continent is 70.6 million metric tons, which accounts only for 7% of the global production (http://www.fao.org). Lack of congruence between the proportion of production and the cultivated area is due to the low productivity of maize in Africa (<2.0 t ha^−1^) as compared to a global average of 5.6 t ha^−1^. In Sub-Sahara Africa (SSA), maize is the primary source of calories (466.5 kcal/capita/day) and is the second most important source of protein (12 g/capita/day) only after wheat. In Ethiopia, maize is the second most popular staple crop after tef (*Eragrostis tef* (Zucc.) Trotter)^2^ with huge potential to feed over 100 million people in the country. Between 2008 and 2017, the total maize production and average grain yield in Ethiopia have increased from 3.8 to 8.1 million tons and from 2.1 to 3.7 t ha^−1^, respectively (http://www.fao.org).

Maize is broadly divided into temperate, subtropical and tropical germplasm depending on latitudinal variations and environmental characteristics^3^. Tropical maize is further classified into lowland, midaltitude and highland. Highland maize germplasm encompasses a wide range of cold tolerant genotypes evolved in Mexico, Guatemala, the Andean highlands and other small patches of cold valleys and mountains^4^. However, they tend to be susceptible to lodging, have taller plants as well as ear heights (the height of a plant from the ground level to the upper most ear-bearing node), sensitive to deep planting, susceptible to inbreeding depression, slow grain drying after harvest for storage and have low harvest index. The International Maize and Wheat Improvement Center (CIMMYT) started highland maize breeding program in Mexico in the 1970s with the intention of developing high yielding and cold tolerant improved germplasm from pools and populations carrying tropical and subtropical genetic backgrounds^5^.

Although maize breeding for the east African highlands started in the 1950s by assembling locally available germplasm and making synthetic populations, the introduction of Ecuador 573 in 1959 to the region significantly impacted highland maize improvement^4^. Ecuador 573 together with Kitale Synthetic II (Kitale-SYN) were used in the reciprocal recurrent selection for genetic improvement and variety development in the African highlands^4,6^. In the 1980s, CIMMYT introduced a tropical highland transition zone adapted pool (Pool 9A) to Africa. The pool was not only made available to farmers as an open-pollinated variety but also was used in various breeding programs^4^. The original Pool 9A was improved for maize streak virus (MSV) resistance at CIMMYT breeding hub in Harare (Zimbabwe), which was then extensively used in highland maize breeding in Africa. The initiation of Highland Maize Genepool Project in 1997 by CIMMYT, in collaboration with the National Agricultural Research Systems (NARS) in eastern Africa, further strengthened the highland maize breeding efforts in the region through introduction and improvement of highland adapted maize germplasm^6^.

Various studies were conducted to determine the genetic diversity, relationship, population structure and heterotic grouping of maize inbred lines developed by CIMMYT^7–13^ and International Institute of Tropical Agriculture (IITA)^9,14–16^ using different genotyping platforms and marker density. Recently, Ertiro *et al*. (2017) genotyped 265 inbred lines developed by EIAR, CIMMYT, and IITA that are widely used in the mid-altitude sub-humid maize agro-ecology of Ethiopia with 220,878 SNPs. The authors reported that only 22% of the inbred lines were considered genetically pure with >95% homogeneity (genetic purity), which requires purification or further inbreeding except those lines deliberately maintained at early inbreeding level to avoid inbreeding depression. Pairwise genetic distances among the 265 inbred lines varied from 0.011 to 0.345, with only <1% of the pairs of lines differing by less than 20% of the total number of scored alleles. Finally, the different multivariate methods consistently suggested the presence of three groups, which generally agreed with pedigree information (genetic background). However, little is known about the genetic purity, variation and population structure of the maize germplasm adapted to the African highlands, which is widely used in eastern Africa. Previous genetic diversity studies conducted on highland maize inbred lines adapted to the African ecology were based on a small number of samples and low marker density^17–21^. For example, Beyene *et al*. (2006 a, b) studied genetic diversity and relationships among 62 Ethiopian highland maize collections using only 20 simple sequence repeat (SSR) markers and eight amplified fragment length polymorphism (AFLP) primers. Legesse *et al*. (2007) assessed the genetic diversity of 35 highland inbred lines from CIMMYT-Ethiopia and 21 mid-altitude inbred lines from CIMMYT-Zimbabwe using 27 SSR markers and nine AFLP primers. Abakemal *et al*. (2015) studied genetic purity and patterns of relationships among 36 maize inbred lines adapted to African highland agro-ecology using 25 SSR markers.

Selective sweeps leave distinct signatures in genomes, which are indicative of loci that have undergone selection^22–24^. Selection increases the frequency of a beneficial allele within a group or population and may even lead to fixation, which then increases the fitness of the individuals carrying it but reduces overall genetic diversity in specific regions that undergone selection^25–27^. Although all the highland-adapted inbred lines have undergone selection for better adaptation to the highland agro-ecology, we expect differential selection in response to target traits, including germplasm type (normal vs. quality protein maize, QPM), heterotic grouping, and abiotic and biotic stresses. Different statistical methods are available to identify genomic regions that have undergone selective sweep^25,28,29^. Therefore, the present study was carried out to (i) assess the genetic purity, genetic relationship and population structure of African highland adapted maize inbred lines using high-density genotyping by sequencing (GBS); (ii) identify genomic regions that have undergone selective sweeps, and examine if those selective sweeps showed greater reduction of nucleotide diversity in specific categorical variables (groups or populations) than others; and (iii) compare the extent of molecular diversity indices and genetic differentiation among different groups of highland maize germplasm.

## Methods

### Plant materials and genotyping

A total of 298 white-grained inbred lines from CIMMYT and Ethiopian Institute of Agricultural Research (EIAR) collaborative highland maize breeding program were used in the study (Supplementary Table S1). These inbred lines are currently widely used in maize breeding programs in the high-altitude sub-humid maize growing areas of eastern and southern Africa (ESA). Early generation lines were originally introduced from CIMMYT-Mexico highland breeding program and CIMMYT-Zimbabwe mid-altitude breeding program, screened under the local environments, and advanced through generations at the EIAR experimental station in Ambo, Ethiopia. Extensive field evaluations were then conducted on advanced generation lines in collaboration with NARS in Kenya, Tanzania, Uganda, Rwanda and Burundi^6^. The lines were selected for desirable agronomic performances, resistance to common leaf rust, *Turcicum* leaf blight, gray leaf spot, and germplasm type (normal or QPM). The inbred lines used in our study comprised of 111 normal endosperm (non-QPM) lines derived primarily from Kitale Synthetic II (Kitale-SYN), Ecuador 573, and Pool 9A. The remaining 187 samples were QPM lines that were either developed through backcross breeding^30^ or extracted from adapted QPM populations. CML144, CML159, and CML176 were the QPM donor parents. Heterotically, 123, 95 and 11 inbred lines belong to groups A, B and AB, respectively, while the remaining 69 inbred lines are not yet assigned.

For each inbred line, seed samples were obtained from Ambo Research Center, Ethiopia. The detailed procedures on genomic DNA extraction, SNP genotyping using GBS^31^ and data filtering were described in a previous study^32^. The 298 inbred lines were genotyped with 955,690 SNPs by the Institute of Biotechnology, Cornell University, the USA, of which 237,018 SNPs (hereafter referred as Dataset-1) with a minor allele frequency (MAF) of ≥0.05 and a maximum missing data of 20% (Table 1) were selected. Dataset-1 was imputed using Beagle V4.2^33^ with the default parameters (i.e., window = 50,000, overlap = 3,000; niterations = 15, and cluster = 0.0) and then filtered if there were SNPs with a MAF less than 0.05, which resulted in 235,019 SNPs (Dataset-2) for further statistical analyses.

**Table 1:** Summary of the different datasets, chromosomal distribution and physical map length of SNP markers used in the present study.

Chromosome	Dataset-1 (unimputed)				Dataset-2 (Imputed)			Dataset-3	Dataset-4
	No. of SNPs	Proportion of SNPs (%)	Map length (Mb)	Proportion of missing data	No. of SNPs	Proportion of SNPs (%)	Map length (Mb)	No. of SNPs	No. of SNPs
Chr 1	36,988	15.6%	301.3	6.8%	36,694	15.6%	301.3	3,516	133
Chr 2	28,793	12.1%	237.0	7.0%	28,567	12.2%	237.0	2,604	0
Chr 3	27,156	11.5%	232.1	7.2%	26,917	11.5%	232.1	2,463	73
Chr 4	22,037	9.3%	241.4	7.3%	21,847	9.3%	241.4	2,458	75
Chr 5	27,767	11.7%	217.7	7.3%	27,483	11.7%	217.7	2,413	46
Chr 6	19,305	8.1%	169.1	7.5%	19,137	8.1%	169.1	1,813	55
Chr 7	20,339	8.6%	176.8	7.4%	20,170	8.6%	176.8	1,892	318
Chr 8	20,730	8.7%	175.7	7.3%	20,566	8.8%	175.7	2,006	151
Chr 9	17,783	7.5%	156.5	7.2%	17,641	7.5%	156.5	1,734	59
Chr 10	16,120	6.8%	150.1	7.4%	15,997	6.8%	150.1	1,601	34
Total	237,018	100.0%	2057.6	—	235,019	100.0%	2,057.6	22,500	944
Mean	23,702	10.0%	205.8	7.2%	23,502	10.0%	205.8	2,250	94

### Statistical analyses

We first computed identity-by-state (IBS)-based genetic distance matrices from both Dataset-1 (unimputed) and Dataset-2 (imputed) and compared the two distance matrices using Mantel test^34^, which showed perfect positive correlation (r = 0.999). All statistical analyses except the model-based population structure were, therefore, computed on Dataset-2. The proportion of heterogeneity, relative kinship coefficients, IBS-based genetic distance matrices, and principal component analysis (PCA) were computed (from Dataset-2) using TASSEL v.5.2.51. Cluster analysis was performed on the genetic distance matrix using the neighbor-joining algorithm implemented in molecular evolutionary genetics analysis (MEGA) v.7.0^35^. The first two principal components (PCs) from the PCA were plotted for visual examination in XLSTAT 2012 (Addinsof, New York, USA; www.xlstat.com) using categorical variables, which include heterotic groups, germplasm type (QPM vs. non-QPM), genetic background and group membership predicted both from population STRUCTURE and cluster analyses.

HapMap format of Dataset-2 was exported to PHYLIP interleaved format using TASSEL v.5.2.51, which was then converted to both MEGA^36^ and ARLEQUIN v.3.5.2.2^37^ formats using PGDSpider v.2.1.1.3^38^. We used MEGA X^36^ to estimate the number of segregating sites (S), the proportion of polymorphic sites (Ps), Theta (θ_S_), nucleotide diversity (θπ) and Tajima’s D test statistic^39^. Analysis of molecular variance (AMOVA)^40^ and F_ST_-based pairwise genetic distance matrices^41^ were computed among categorical variables using ARLEQUIN v.3.5.2.2^37^. *F*_*ST*_ values between pairs of populations or groups are indicative of the evolutionary processes that influence the structure of genetic variation with <0.05, 0.05–0.15, 0.15–0.25 and >0.25 indicating little, moderate, great and very great genetic differentiation, respectively^42^. To minimize the computational requirement in population structure analyses, the 235,019 SNPs in Dataset-2 were further filtered using a MAF of 0.10 and a minimum physical distance of 10-kb between adjacent markers, which resulted in 22,500 SNPs (hereafter referred as Dataset-3). Population structure was analyzed using Dataset-3 and the model-based method implemented in the software package STRUCTURE v.2.3.4^43^ as described in our previous studies^32,44^. Inbred lines with membership probabilities >60% were assigned to the same clusters, while those with probabilities <60% in any group were assigned to a “mixed” group.

SweeD v.4.0.0^45^ was used to detect selective sweeps that may have undergone selection during breeding process. For this purpose, Dataset-2 was converted into reference and alternative alleles using the variant call format (VCF) option in TASSEL v.5.2.51, which corresponds to the major and minor alleles, respectively. SweeD v.4.0.0 was run on the VCF input file as described in a previous study^45^ to evaluate a grid of 10,000 equidistant physical locations. The threshold score for declaring selective sweeps was set as the 99.9%, so the 0.1% with likelihood scores >3.1 were retained to represent a candidate selective sweep. The start and end of physical positions of each selective sweep region were used to search for candidate genes and their predicted functions^46^ at the Gramene Genome Brower (http://ensembl.gramene.org/Zea_mays/Info/Index).

## Results

### Marker summary and genetic purity

Among the 955,690 SNPs initially generated through GBS, about 25% of the SNPs were used for statistical analyses. The 235,019 SNPs in Dataset-2 were distributed across all 10 chromosomes, which varied from 15,997 on chromosome 10 to 36,694 SNPs on chromosome 1 (Table 1). Minor allele frequency per SNP ranged from 0.05 to 0.50, with an overall average of 0.233 (Supplementary Table S2a). Genetic purity estimated per inbred line ranged from 67.9% to 99.8% (Supplementary Fig. S1, Supplementary Table S1), with a mean of 88.9%. Because of the low genetic purity previously reported in most inbred lines adapted to the Ethiopian mid-altitude sub-humid maize agro-ecology of Ethiopia^32^, we increased the threshold value from 5.0% to 6.25%, which is the expected average residual heterozygosity (heterogeneity) for lines developed after four generation of inbreeding. Using this threshold criterion, only 34 of the 298 inbred lines (11.4%) were considered fixed with a heterogeneity of ≤6.25, while 57.7% and 30.8% of the inbred lines had heterogeneity varying from 6.26 to 12.50 and from 12.51 to 32.10%, respectively (Supplementary Table S1).

### Genetic relatedness and distance

**Figure 1:**
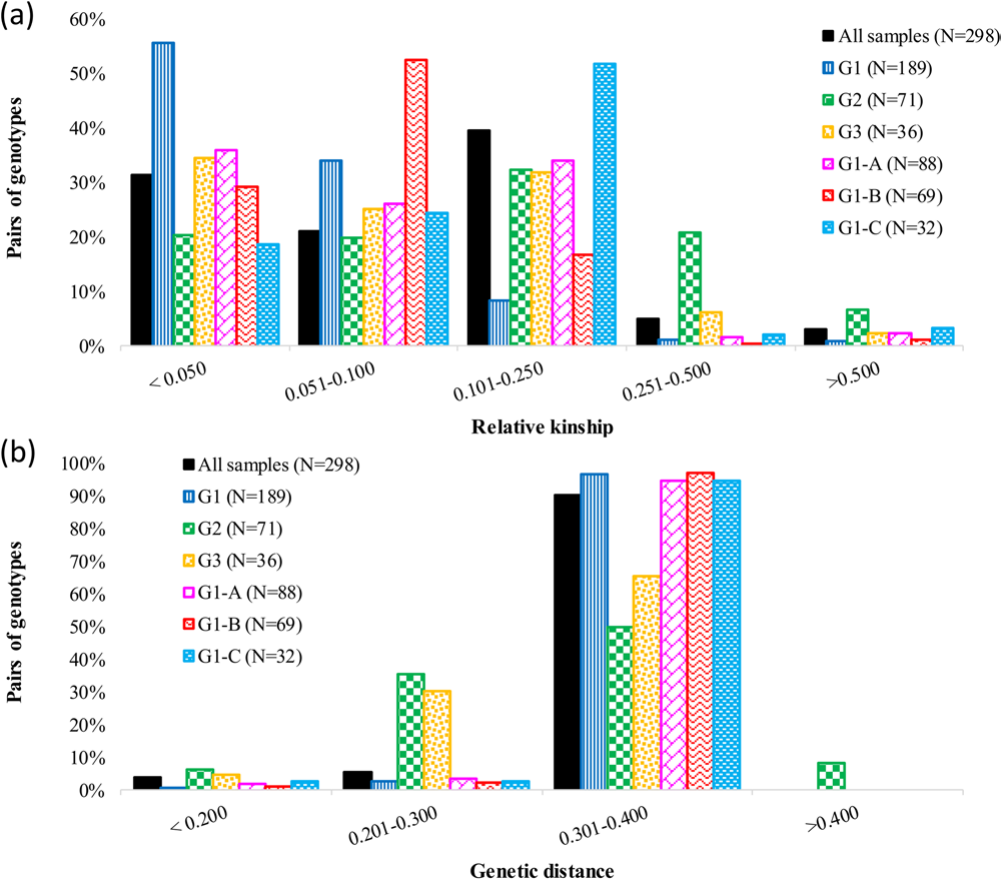
Frequency distribution of (**a**) relative kinship and (**b**) pairwise genetic distance matrices computed using SNPs that were polymorphic within a given number of inbred lines, each with a minor allele frequency >0.05: (i) all the 298 inbred lines using 235,019 SNPs; (ii) 88 inbred lines that belong to Group-1A (G1-A) using 218,208 of 235,019 SNPs; (iii) 69 inbred lines in G1-B using 214,566 of 235,019 SNPs; (iv) 32 inbred lines in G1-C using 200,864 of 235,019 SNPs; (v) 71 inbred lines in G2 using 129,031 of 235,019 SNPs; (vi) 36 inbred lines in G3 using 171,163 of 235,019 SNPs.

Kinship coefficients between pairs of the 298 inbred lines ranged between 0.00 and 1.98 (on a scale of 0 to 2). Nearly 32% of the pairwise relative kinship values were close to zero (<0.05), 66% were between 0.051 and 0.500 and the remaining 3% between 0.501 to 1.98 (Supplementary Table S3a). When kinship values were compared among groups predicted based on cluster analysis and the model-based STRUCTURE (see below), only 20.3% of the pairs of inbred lines in Group-2 had values close to zero as compared to 34.6% in Group-3 and 55.7% in Group-1 (Fig. 1, Table S3). Genetic distance between pairs of inbred lines ranged from 0.010 to 0.360 (Supplementary Table S4), and the overall mean was 0.323. Nearly 91% of the pairs of 298 lines had genetic distance values between 0.301 and 0.360 as compared to just 0.3% of the pairs that differed by <10% of the scored alleles. About 58.4% and 65.4% of the pairs of inbred lines belonging to Group-2 and Group-3, respectively, differed by >30% of the scored alleles (0.301–0.400) as compared to the 94.6–97.0% pairs in Group-1 predicted based on cluster analysis and model-based population structure (Fig. 1, Supplementary Table S4).

### Population structure and genetic relationship

**Figure 2:**
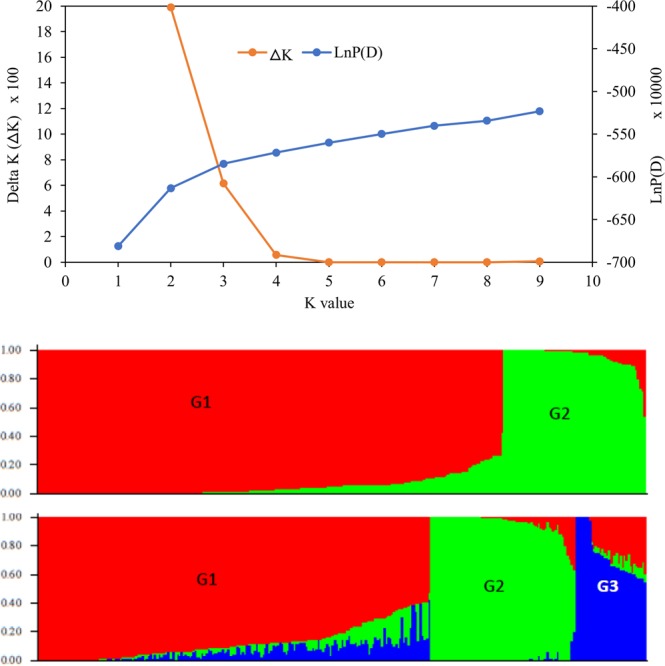
Population structure of 298 maize inbred lines based on 22,500 SNPs in Dataset-3: (**a**) plot of LnP(D) and a ΔK calculated for K ranging from 1 to 10, with each K repeated thrice; (**b**) population structure of the 298 inbred lines at K = 2 and K = 3. Every line is represented by a single vertical line that is partitioned into K colored segments on the x-axis, with lengths proportional to the estimated probability membership (y-axis) to each of the K inferred clusters. For membership of each line, see Supplementary Table S1.

The log probability of the data (LnP(D)) and *ad hoc* statistics ∆K obtained from the model-based population structure analysis suggested that the 298 lines can be divided into two or three possible groups or sub-populations (Fig. 2). However, when the results at various K values were compared with their pedigree information and breeding history, the groups obtained at K = 3 were considered as the best possible number of groups. The proportion of inbred lines assigned to Group-1, Group-2, and Group-3 was 64%, 23%, and 12%, respectively, with only two lines belonging to a mixed group (Table 2, Supplementary Table S1). The first group consisted of 192 inbred lines with mixed heterotic groups, genetic background, and endosperm modification. The second group consisted of 69 QPM inbred lines from heterotic group A (68 lines) and B (1 line) that were developed using CML144 as donor parent and Ecuador-573 (55 lines), Pool 9A-SR (13 lines) and Kitale-SYN (1 line) as recurrent parents. The third group consisted of 35 non-QPM inbred lines extracted from Pool 9 A.

**Table 2:** Summary of the 298 inbred lines assigned to the three groups predicted based on the model-based population structure analysis by heterotic grouping, endosperm modification (kernel type) and genetic backgrounds.

Category	Group	Group-1	Group-2	Grop-3	Mixed	Sub-total
Heterotic groups	A	53	68		2	123
	AB	11				11
	B	92	1	2		95
	Unknown	36		33		69
	**Total**	**192**	**69**	**35**	**2**	**298**
Neighbor-joining cluster analysis	G1-A	88				88
	G1-B	69				69
	G1-C	32				32
	G2		69		2	71
	G3	1		35		36
	Ungrouped	2				2
	**Total**	**192**	**69**	**35**	**2**	**298**
Germplasm type	Non-QPM	75		35	1	111
	QPM	117	69		1	187
	**Total**	**192**	**69**	**35**	**2**	**298**
Genetic background of recurrent parents	Ecuador-573	14	55			69
	Kitale-SYN	29	1			30
	Others	11				11
	Pool-9A	9		35		44
	Pool-9A-SR	65	13		2	80
	Pop-502-SR	17				17
	SADVLA	10				10
	SUSUMA	24				24
	Tuxpeno	13				13
	**Total**	**192**	**69**	**35**	**2**	**298**
Genetic background by donor parents	CML144	29	69		1	99
	CML159	15				15
	CML176	46				46
	Non-CML	102		35	1	138
	**Total**	**192**	**69**	**35**	**2**	**298**

**Figure 3:**
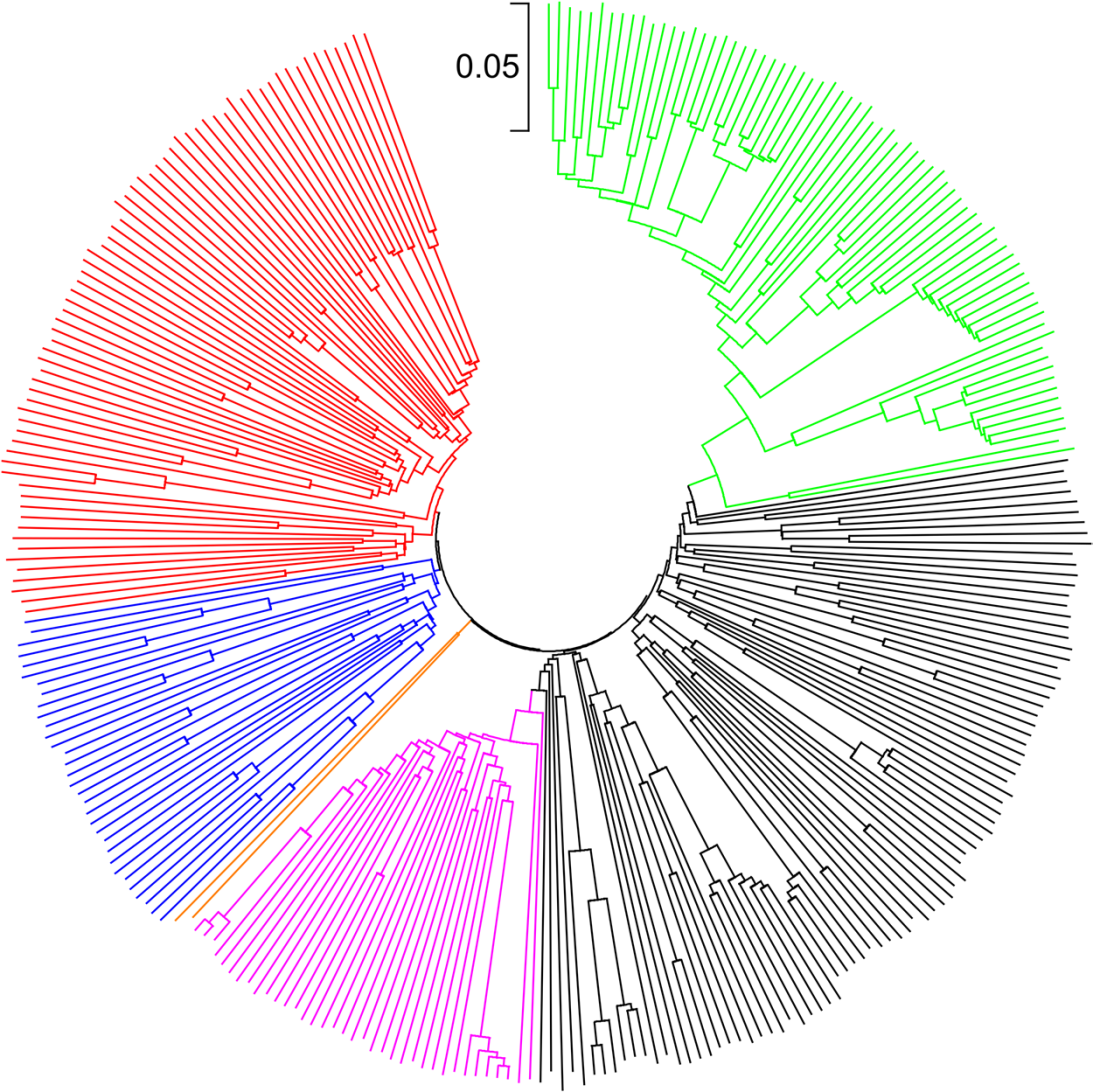
Neighbor-joining tree of 298 inbred lines based on identity-by-state genetic distance matrix computed from 235,019 SNPs, each with minor allele frequency >0.05. Line colors are as follows: Group-1A (black); Group-1B (red), Group-1C (blue), Group-2 (green), Group-3 (pink) and ungrouped (orange). Group-1, Group-2, and Group-3 were obtained based on the model-based STRUCTURE. See Supplementary Table S1 for details of each group membership.

The neighbor-joining (NJ) tree constructed from the genetic distance matrix grouped 296 of the 298 inbred lines into three major groups as the model-based STRUCTURE and five sub-groups; two inbred lines were not assigned into any of the sub-groups (Fig. 3, Supplementary Table S1). Nearly all inbred lines that belong to Group-2 and Group-3 remained the same as the group membership predicted based on the model-based population structure analysis. On the other hand, lines belonging to the first group in the model-based population structure were further divided into three subgroups (Group-1A, Group-1B, and Group-1C) in the cluster analysis (Supplementary Table S1). Group-1A consisted of a total of 88 inbred lines of mixed heterotic groups, germplasm type (both QPM and non-QPM), and genetic backgrounds. Group-1B had 69 inbred lines, which are primarily QPM (65 lines) with mixed heterotic groups and diverse genetic backgrounds, while Group-1C consisted of 32 inbred lines that were primarily non-QPM with both Ecuador-573 and Kitale-SYN genetic background, but mixed heterotic groups (Supplementary Table S1). As shown in Fig. 3, however, the three sub-groups clustering pattern based on the model-based population structure analysis does not fully match the pattern obtained in NJ analysis.

**Figure 4:**
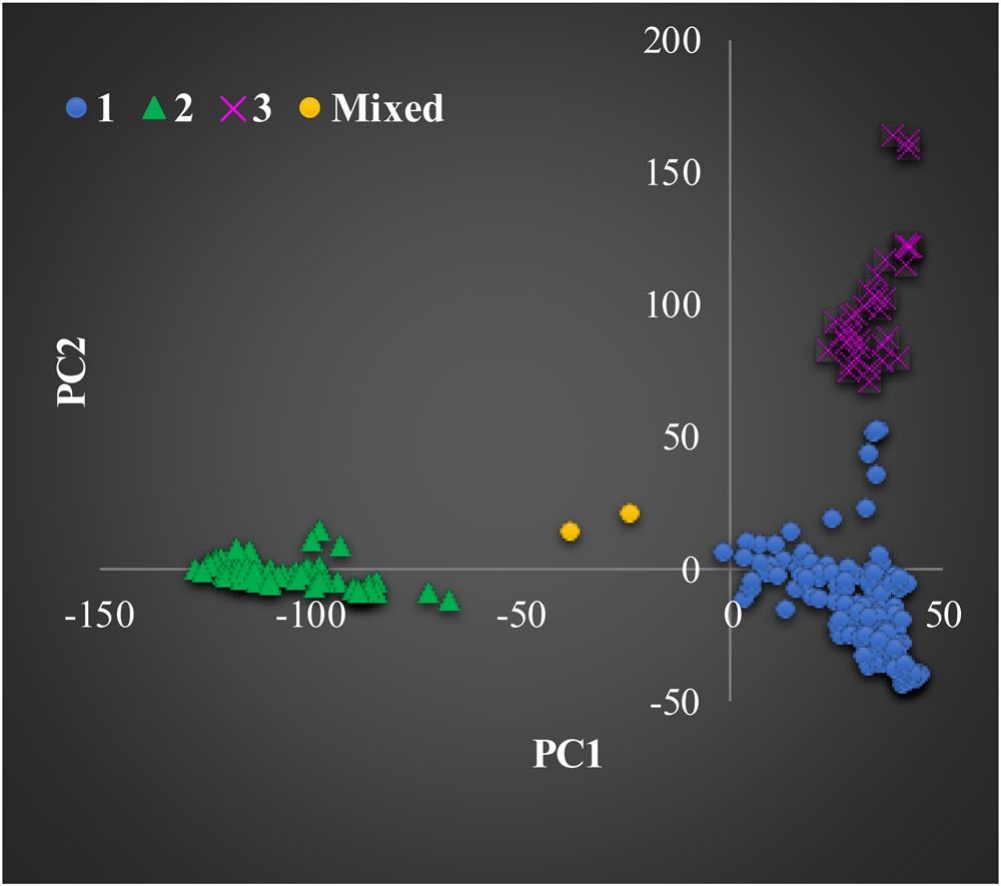
Plot of PC_1_ (11.3%) and PC_2_ (5.4%) from a principal component analysis of 298 inbred lines using 235,019 SNPs, each with minor allele frequency >0.05. Group-1 (blue), Group-2 (green), Group-3 (pink) and mixed (orange) were obtained from the model-based STRUCTURE at K = 3. See Supplementary Table S1 for details of each group membership.

To get insight on the patterns of relationship among the 298 inbred lines, we constructed various phylogenetic trees (Fig. 3, Supplementary Fig. S2) and also plotted PC1 (16.9%) against PC2 (8.3%) from PCA using diverse categorical variables (Fig. 4, Supplementary Fig. S3), including heterotic grouping, germplasm type (QPM vs. non-QPM), genetic backgrounds and predicted group memberships based on both cluster and model-based STRUCTURE. The different plots clearly demonstrated three distinct groups, which was consistent with the group membership of the model-based STRUCTURE at K = 3 than any of the other categorical variables. Nearly 77% of the 298 inbred lines have already been assigned to heterotic groups A (123 lines), B (95 lines) and AB (11 lines) by breeders based on combining ability tests, mainly using diallel and line-by-tester analyses. As shown in Supplementary Figs S2 and S3, lines belonging to the same heterotic group did not necessarily clustered together. Nearly 95% of the inbred lines belonging to heterotic group B showed clear population structure as compared to those in heterotic group A that were divided into two subgroups.

### Genetic differentiation

Results from the partitioning of the molecular variance by different categorical variables revealed that differences in heterotic groups (A vs. B) and germplasm type (QPM vs. non-QPM) accounted for 12.0% and 8.1% of the genetic variation, respectively, which both fell under moderate genetic differentiation. On the other hand, the differentiation among groups based on genetic background (pedigree information), groups predicted based on cluster analysis and the model-based population structure accounted for 18.8–21.6% and 25.3–29.6% of the total molecular variation, respectively (Table 3), which suggest great and very great genetic differentiation. When pairwise *F*_ST_ values between groups were compared (Supplementary Table S5), the values among groups predicted from the model-based STRUCTURE was the highest between Group-2 and Group-3 (0.498) and the lowest between the Group-1 and Group-3 (0.221), which is also evident in the PCA plot (Fig. 4). F_ST_ values of the 21 possible pairwise comparisons based on the genetic backgrounds of the recurrent parents varied from 0.086 between Kitale-SYN and Pool-9A to 0.368 between Ecuador-573 and Pop-502-SR with most pairs showing either moderate (0.05–0.15) or great (0.15–0.25) genetic differentiation.

**Table 3:** Analysis of molecular variance (AMOVA) of 298 inbred lines grouped on different categorical variables for the extraction of SNP variation among and within groups (populations) based on 235,019 SNPs.

Category	Source of variation	Degree of freedom	Sum of squares	Variance components	Percentage of variation
Groups based on STRUCTURE at K = 3	Among groups	2	1,396,725.3	8,954.0	29.6
	Within groups	293	6,233,282.8	21,274.0	70.4
	Total	295	7,630,008.0	30,228.0	100.0
Groups based on neighbor-joining method	Among groups	4	1,676,030.9	6,964.1	25.3
	Within groups	291	5,969,328.7	20,513.2	74.7
	Total	295	7,645,359.6	27,477.3	100.0
Groups based on genetic backgrounds of recurrent parents	Among groups	6	1,194,314.2	5,066.3	18.8
	Within groups	280	6,144,305.6	21,943.9	81.2
	Total	286	7,338,619.8	27,010.2	100.0
Groups based on genetic backgrounds of recurrent parents, excluding “Others” in Table 2	Among groups	7	1,495,876.8	5,759.3	21.6
	Within groups	279	5,842,743.0	20,941.7	78.4
	Total	286	7,338,619.8	26,701.0	100.0
Groups based on genetic backgrounds of CML donor parents	Among groups	2	509,151.8	5,532.8	20.2
	Within groups	157	3,436,444.4	21,888.2	79.8
	Total	159	3,945,596.1	27,421.0	100.0
Heterotic groups	Among groups	1.0	371,556.3	3,244.0	12.0
	Within groups	216.0	5,139,101.0	23,792.1	88.0
	Total	217.0	5,510,657.3	27,036.1	100.0
Groups based on germplasm type	Among groups	1	330,070.6	2,191.0	8.1
	Within groups	296	7,353,918.8	24,844.3	91.9
	Total	297	7,683,989.5	27,035.3	100.0

### Diversity indices and selective sweeps

**Figure 5:**
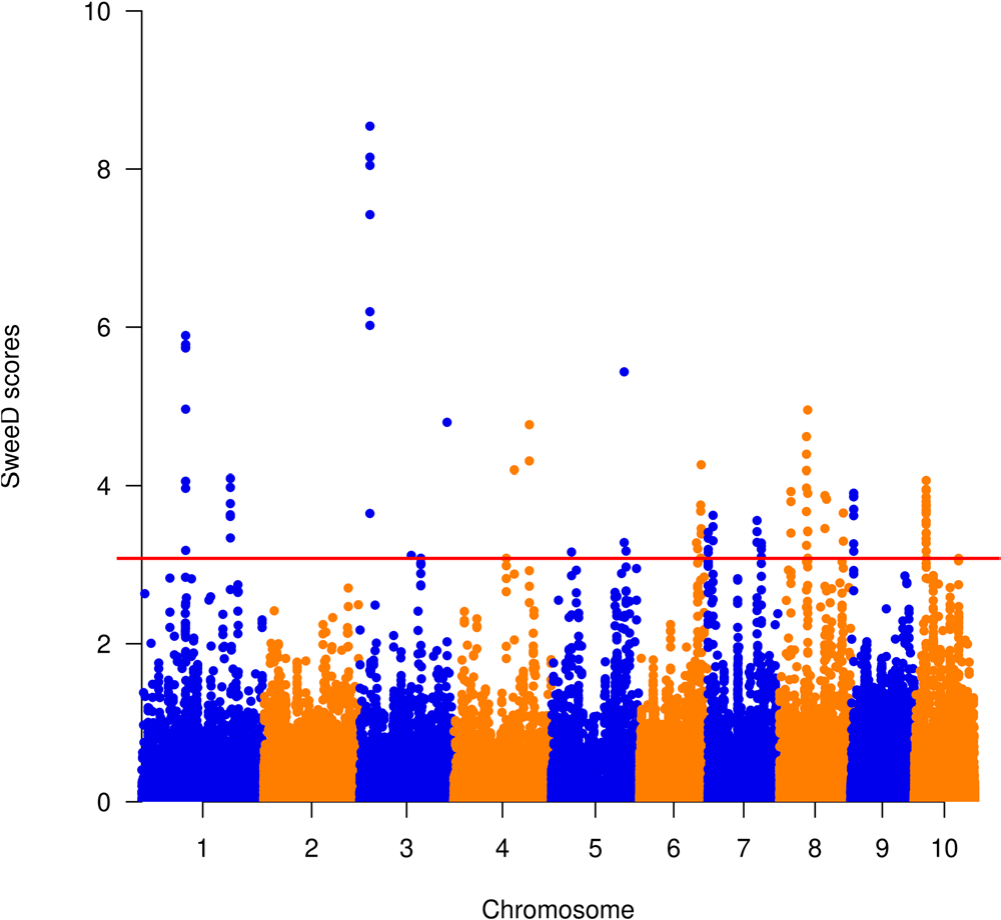
Manhattan plot showing the 22 selective sweep regions detected using SweeD v.4.0.0. The horizontal solid line indicates the threshold value of 3.1 for declaring candidate selective sweeps.

Table 4 summaries the marker polymorphism, diversity indices, and Tajima’s D computed for inbred lines belonging to the same categorical variables (heterotic groups, germplasm type, genetic backgrounds and predicted group membership based on NJ cluster analysis and the model-based STRUCTURE). Of the 235,019 segregating sites across the 298 inbred lines, the number of segregating sites, proportion of polymorphic sites and nucleotide diversity (π) observed within Group-2 and Group-3 predicted based on the model-based STRUCTURE and NJ cluster analyses were much smaller than Group-1, which all indicate reduction in diversity in the former two groups. Inbred lines with Pop-502-SR, SADVLA, SUSUMA, and Tuxpeno genetic backgrounds showed smaller diversity indices than those lines derived from Ecuador-573, Kitale-SYN, Pool-9A and Pool-9A-SR. However, we did not observe obvious differences when the analyses were conducted using the two heterotic groups (A vs. B) and germplasm type (QPM vs. non-QPM) as categorical variables (Table 4). Tajima’s D values computed from Dataset-2 were negative in both Group-2 and Group-3, which is an indication for stronger positive selection in these two groups than Group-1. SweeD^45^ identified 22 candidate selective sweep regions distributed across all chromosomes except chromosome 2 (Table 5, Fig. 5). The selective sweep regions spanned from 6-kb to 4,229-kb and consisted of clusters of markers that varied from 8 to 125 SNPs, except one region on chromosome 8 (Chr8-Reg-02) that had just one SNP (Table 5). Overall, a total of 944 SNPs were mapped within the 22 selective sweep regions (Dataset-4). Selective sweeps increase the frequency of beneficial alleles and surrounding variants and may eventually lead to fixation, while recombination and mutation introduce new alleles that are rare (causing alleles of very low frequency), which are evident in Supplementary Table S2.

**Table 4:** Summary of the molecular diversity indices for different categorical variables based on Dataset-2 (235,019 SNPs) and Dataset-4 (944 SNPs that fell within 22 selective sweeps identified using SweeD). Dataset-4 was used to assess reduction in diversity indices within each group (but not among groups) as compared to the genome-wide SNPs in Dataset-2.

Groups	N*	Dataset-2 (235,019 genome-wide SNPs)**					Dataset-4 (944 SNPs with 22 selective sweeps)**				
		*S*	*Ps*	*Θ*_*S*_	*Θ*_*π*_	*D*	*S*	*Ps*	*Θ*_*S*_	*Θ*_*π*_	*D*
**Groups based on TRUCTURE at K** = **3**											
Group-1	192	234,655	0.998	0.171	0.212	0.765	942	0.998	0.171	0.140	−0.595
Group-2	69	133,423	0.568	0.118	0.114	−0.119	283	0.300	0.062	0.052	−0.570
Group-3	35	172,317	0.733	0.178	0.143	−0.747	442	0.468	0.114	0.069	−1.492
**Groups based on NJ cluster analysis**											
Group-1A	88	226,991	0.966	0.191	0.210	0.340	865	0.916	0.181	0.131	−0.965
Group-1B	69	221,508	0.943	0.196	0.185	−0.202	860	0.911	0.190	0.121	−1.269
Group-1C	32	204,603	0.871	0.216	0.215	−0.020	761	0.806	0.200	0.170	−0.590
Group-2	71	145,321	0.618	0.128	0.119	−0.258	305	0.323	0.067	0.054	−0.661
Group-3	36	173,390	0.738	0.178	0.142	−0.770	462	0.489	0.118	0.069	−1.573
**Groups based on endosperm modification**											
QPM	187	233,320	0.993	0.171	0.211	0.754	937	0.993	0.171	0.113	−1.097
Non-QPM	111	229,277	0.976	0.185	0.213	0.514	878	0.930	0.176	0.131	−0.854
**Groups based on genetic background of QPM donor parents**											
CML144	99	205,635	0.875	0.169	0.179	0.201	638	0.676	0.131	0.089	−1.097
CML159	15	170,423	0.725	0.223	0.170	−1.067	487	0.516	0.159	0.108	−1.425
CML176	46	218,615	0.930	0.212	0.207	−0.088	825	0.874	0.199	0.133	−1.217
Non-CML	27	184,241	0.784	0.203	0.156	−0.927	628	0.66525	0.1726	0.10821	−1.4805
**Groups based on genetic background of recurrent parents**											
Ecuador-573	69	197,259	0.839	0.175	0.148	−0.532	653	0.692	0.144	0.074	−1.714
Kitale-SYN	30	206,194	0.877	0.221	0.226	0.084	739	0.783	0.198	0.158	−0.775
Pool-9A	44	191,329	0.814	0.187	0.167	−0.407	596	0.631	0.145	0.092	−1.365
Pool-9A-SR	80	223,315	0.950	0.192	0.209	0.309	850	0.900	0.182	0.122	−1.135
Pop-502-SR	17	153,519	0.653	0.193	0.162	−0.711	470	0.498	0.147	0.107	−1.172
SADVLA	10	140,062	0.596	0.211	0.160	−1.217	398	0.422	0.149	0.102	−1.575
SUSUMA	24	175,493	0.747	0.200	0.151	−0.998	594	0.629	0.169	0.106	−1.499
Tuxpeno	13	156,379	0.665	0.214	0.159	−1.201	426	0.451	0.145	0.096	−1.565
**Groups based on heterotic grouping**											
Heterotic group A	123	226,605	0.964	0.179	0.199	0.369	848	0.898	0.167	0.103	−1.273
Heterotic group B	95	232,840	0.991	0.193	0.207	0.250	921	0.976	0.190	0.132	−1.045
Heterotic group AB	11	171,143	0.728	0.249	0.231	−0.339	489	0.518	0.177	0.158	−0.509
**All inbred lines without groups**											
All samples	298	234,956	1.000	0.159	0.220	1.202	943	0.999	0.159	0.125	−0.682

^*^In cases where the sample size (N) do not add up to 298, some lines were excluded from the selective sweep analyses, which included the following: (i) two lines assigned to a “mixed” group at K = 3 and those remained unassigned to any of the sub-groups in the NJ cluster analysis; (ii) 69 lines with yet unknown heterotic groups; (iii) 11 lines with uncertain recurrent parent genome and (iv) 26 lines with uncertain genetic background of QPM donor parents. See Supplementary Table S1 for details.

**Number of segregating sites (S); Proportion of polymorphic sites (Ps); Theta (θ_S_); Nucleotide diversity (Θπ); Tajima’s D test statistic (D).

**Table 5:** Summary of the 22 selective sweeps identified using SweeD, including chromosomal position, number of SNPs that fell within each region and number of candidate genes. See Supplementary Table S6 for details on candidate genes identified in each region.

Selective sweep name	Selective sweep interval	Chrom.	Minimum likelihood	Maximum likelihood	Minimum Alpha*	Maximum Alpha*	Start position (bp)	End position (bp)	Interval (kb)	No. of SNPs within the selective sweep interval (Dataset-4)	No. of candidate genes
Chr1-Reg-01	1:106060208-110288743	1	3.2	5.9	3.9E-06	4.5E-05	106,060,208	110,288,743	4229	125	45
Chr1-Reg-02	1:218716421-219612163	1	3.3	4.1	1.6E-05	3.9E-04	218,716,421	219,612,163	896	8	9
Chr3-Reg-01	3:25123538-25922284	3	3.6	8.5	2.5E-05	1.1E-04	25,123,538	25,922,284	799	22	14
Chr3-Reg-02	3:127512961-127518662	3	3.1	3.1	1.7E-04	1.7E-04	127,512,961	127,518,662	6	14	2
Chr3-Reg-03	3:215786813-216005903	3	4.8	4.8	9.9E-05	9.9E-05	215,786,813	216,005,903	219	37	4
Chr4-Reg-01	4:149724977-149899895	4	4.2	4.2	6.4E-05	6.4E-05	149,724,977	149,899,895	175	34	1
Chr4-Reg-02	4:186912590-187083281	4	4.3	4.8	1.1E-04	1.4E-04	186,912,590	187,083,281	171	41	6
Chr5-Reg-01	5:49802255-49836436	5	3.2	3.2	1.3E-04	1.3E-04	49,802,255	49,836,436	34	11	0
Chr5-Reg-02	5:179785894-179940120	5	3.3	5.4	9.5E-05	5.5E-04	179,785,894	179,940,120	154	9	1
Chr5-Reg-03	5:184394549-184699627	5	3.2	3.2	9.8E-05	9.8E-05	184,394,549	184,699,627	305	26	6
Chr6-Reg-01	6:140675365-140726905	6	3.2	3.3	8.4E-05	2.6E-04	140,675,365	140,726,905	52	17	1
Chr6-Reg-02	6:150939481-151135404	6	3.4	4.3	4.7E-05	6.8E-04	150,939,481	151,135,404	196	38	4
Chr7-Reg-01	7:32523-1269208	7	3.2	3.4	1.8E-05	2.2E-05	32,523	1,269,208	1237	119	32
Chr7-Reg-02	7:11953168-13065716	7	3.3	3.6	2E-05	2.4E-05	11,953,168	13,065,716	1113	28	23
Chr7-Reg-03	7:120966174-121400969	7	3.3	3.6	5.8E-05	1.2E-04	120,966,174	121,400,969	435	71	9
Chr7-Reg-04	7:131185931-132315208	7	3.2	3.3	2E-05	8.2E-05	131,185,931	132,315,208	1129	100	20
Chr8-Reg-01	8:27653451-28591523	8	3.4	3.9	2.5E-05	1.0E-04	27,653,451	28,591,523	938	35	15
Chr8-Reg-02	8:65750670-67244274	8	3.2	5.0	1E-05	3.0E-04	65,750,670	67,244,274	1494	1	25
Chr8-Reg-03	8:111704655-112150258	8	3.5	3.9	2.9E-05	9.8E-05	111,704,655	112,150,258	446	28	3
Chr8-Reg-04	8:157235205-157836943	8	3.3	3.7	3.2E-05	3.8E-05	157,235,205	157,836,943	602	87	21
Chr9-Reg-01	9:7105365-7663379	9	3.2	3.9	2E-05	6.3E-05	7,105,365	7,663,379	558	59	15
Chr10-Reg-01	10:29222096-30868221	10	3.2	4.1	1E-05	7.9E-05	29,222,096	30,868,221	1646	34	9

^*^The minimum and maximum alpha values are given in exponentials (e.g., 3.9E-06 = 3.9 × 10^−6^).

In dataset-4, the major and minor allele frequency of the 944 SNPs that fell within the selective sweeps were 0.800-0.950 and 0.05-0.200, respectively. Nearly 3%, 72%, 53% and 37% of the 944 SNPs had major allele frequency greater than 0.950 in Group-1, Group-2, Group-3 and Group-2 and Group-3 combined, respectively. In fact, 32% and 17% of the 944 SNPs were fixed in Group-2 and Group-3, respectively, as compared to none in Group-1. Such results suggest that most SNPs that fell within the 22 selective sweep regions showed a reduction in diversity in Group-2 and Group-3, which is likely due to selection for better adaptation to specific traits that may not be the case in Group-1. Comparisons of Ps and θ_S_ computed from the 944 SNPs (Dataset-4) that fell within the 22 selective sweeps with the genome-wide SNPs (Dataset-2) showed reduction in both Group-2 and Group-3 than Group-1. A less obvious reduction were noted among inbred lines belonging to the different heterotic groups, germplasm type and genetic backgrounds (Table 4). To gain insight into possible roles of each of the selective sweep region, we compiled a list of 265 protein-coding candidate genes that fell within the 21 of 22 selective sweep regions (Supplementary Table S6). Each of the 21 selective sweeps consisted of one to forty-five protein-coding genes. Some of the candidate genes had known functions, which are summarized in Supplementary Table S6 and partly discussed in the next section.

## Discussion

### Genetic purity in African highland maize inbred lines

Maintenance of genetic purity in inbred lines by minimizing residual heterozygosity (heterogeneity) is important for quality seed production^32,47,48^. The threshold value may vary depending on the purpose of the line development program and level of inbreeding. In the current study, only 34 of the 298 inbred lines (~11%) were found to be genetically homogeneous (Supplementary Table S1), which agrees with another recent study on maize inbred lines adapted to the mid-altitude sub-humid maize ecology in Ethiopia^32^. In that previous study, about 53% of the maize inbred lines developed by EIAR showed higher than expected level of genetic heterogeneity as compared to 13% and 8% of the inbred lines developed by CIMMYT and IITA breeding programs, respectively, which may be due to one or more of the following reasons. First, the three institutions use different methods for line maintenance, besides the source germplasm. EIAR breeders often use sib-mating (by bulking pollens of multiple plants from the same entry) during line development, which is less common both at CIMMYT and IITA. In addition, the high level of genetic heterogeneity within EIAR maize inbred lines could also be due to human errors (e.g., contamination by off types, stray pollens and/or seed admixture) during line development and/or line maintenance. If such types of errors occur, the sib-mating method is more prone to introducing new sources of genetic variability that in turn reduces genetic purity than selfing of individual plants. Because of the extensive collaboration between CIMMYT-Ethiopia and EIAR, including sharing nurseries, most of the inbred lines analyzed in the present study could have resulted from a combination of sib-mating and selfing.

Second, most of the source germplasm used for developing the inbred lines in the current study were composites, pools, and synthetics^49^, which are suitable for developing open-pollinated varieties (OPVs) but may not be suitable for extracting genetically pure inbred lines. Third, some of the inbred lines were deliberately extracted from early generation (such as S_3_) lines and maintained by sib-mating to avoid severe inbreeding depression upon continuous self-pollination^4^. Although such approach is useful to attain higher seed yield per unit area, which in turn decreases the cost of seed production and increases access to seed by small-scale farmers^50^, it would be very challenging in terms of line maintenance. However, the third case is a less likely scenario as there are multiple lines with heterogeneity greater than 12.5%, which is the expected average heterogeneity among lines extracted at S_3_ generation.

Currently, there is more demand in developing uniform hybrids using genetically pure parental lines, especially doubled haploid lines, as this has several advantages, including better heterosis, simplicity in parental line maintenance and implementing quality control during hybrid seed production^32,47,48,51^. As a result, maize breeders are using fixed lines in their new pedigree starts up and advance each generation through selfing than sib-mating. One of the immediate solutions for improving genetic purity of the inbred lines used in the present study may be to purify seed stocks of those lines with higher than expected heterogeneity by rouging off-types in seed maintenance and production plots, but such method requires enormous efforts and incurs additional costs. The long-term solution is to use doubled haploid (DH) technology in developing genetically pure doubled haploid (DH) lines that can be derived in a short period of time^52–54^. In partnership with the Kenya Agriculture and Livestock Research Organization (KALRO), a state-of-the-art maize DH facility for Africa has been established in 2013 by CIMMYT at Kiboko station, Kenya, which is annually producing nearly 70,000 DH lines from African-adapted maize source germplasm.

### Genetic relationship and population structure

Relative kinship coefficients are widely used as an indicator of the genetic relationship between pairs of genotypes, where values close to zero indicate a lack of relationship, while higher values indicating stronger relationship. About sixty-nine percent of the pairwise comparisons of the 298 inbred lines had kinship values ranging from 0.05 to 1.98 as compared to just 32% that had kinship values close to zero, suggesting presence of high level of genetic similarity that may be due to the use of closely related parents that tend to introduce redundant alleles in a breeding program. Similar results were reported in previous studies in maize inbred lines from different breeding programs^7–10,32^. The 32% pairs of highland maize inbred lines with kinship coefficients close to zero was six-fold greater than the 5% reported in maize inbred lines originated from CIMMYT ESA breeding programs^7^, but nearly half of the values reported for maize inbred lines adapted to the mid-altitude ecologies of Ethiopia^32^, the global maize collection^10^, inbred lines from INERA and IITA^14^, inbred lines from CIMMYT and IITA^9^ and CIMMYT maize inbred lines^8^. On the other hand, nearly 91% of the pairs of 298 inbred lines differed by 30–36% of the scored alleles (of 235,019 SNPs in Dataset-2) as compared with just 10% of the pairs that differed by ≤30% of the scored alleles (Supplementary Table S4 and Fig. 1).

The high genetic differences among most pairs of inbred lines agrees with pedigree information and breeding history, as have been reported in other studies^11,14,17,21,32^. Of the inbred lines assigned into heterotic groups based on combining ability tests through diallel and line-by-tester analyses^6^, only part of them showed clear population structure which is expected due to their genetic backgrounds (composites, pools, synthetics). Several previous studies reported the lack of consistencies between heterotic classification based on genotype data and combining ability or pedigree information in tropical maize germplasm^7,8,14,32^. The broad genetic base of the germplasm, lack of clear information on origin and heterotic background, inadequate pedigree information, short breeding history, and use of variable testers and testcross evaluation for assigning lines to heterotic groups have been frequently cited as possible reason for disagreement between markers-based heterotic grouping and combining ability and pedigree-based heterotic assignment.

### Role of candidate genes in selective sweeps

As shown in the Manhattan plot in Fig. 5, the highest SweeD score was 8.5, which was observed within 799-kb interval on chromosome 3 (3:25123538-25922284). This region harbored 14 protein-coding genes, including *glycosyl transferases* in family 61 protein that mediate arabinofuranosyl transfer onto xylan in grasses^55,56^, which plays an essential structural role in cell walls of all plants and valuable components of human and animal nutrition due to its major dietary fiber composition in cereals^57,58^. One of the selective sweeps on chromosome 1 (1:106060208-110288743) consisted of 45 candidate genes, including (i) the *WRKY*-transcription factors that play crucial roles in plant growth and development, defense regulation and response to different biotic and abiotic stresses^59,60^; (ii) *roothairless6* (*rth6)*, which is one of the genes that control root hairs formation and facilitates nutrient uptake and optimal development^61,62^; (iii) *JUMONJI*-transcription factor 14 (*JMJ14*) and *CCAAT*-binding transcription factor that control flowering time^63–65^. The selective sweep on chromosome 5 (5:184394549-184699627) harbors the maize red *aleurone1* (*pr1*) that encodes a *CYP450-dependent flavonoid 3*′*-hydroxylase*, which is required for the biosynthesis of purple and red anthocyanin pigments. Anthocyanins accumulate in maize pericarps, cob glumes, and silks^66^ and believed to have a protective role in plants against extreme temperatures. The *pr1* locus has also been extensively used as a phenotypic marker in determining kernel aleurone color by hydroxylation of anthocyanin compounds^67^. Different studies have implicated members of the *bZIP* family of transcription factors (proteins that bind to the *G-box*) as mediators of abscisic acid dependent gene expression^68,69^ of which *bZIP* transcription factor 1 (*bzip1*) is located in one of the selective sweep regions (3:215786813-216005903) identified in the present study. Abscisic acid plays a central role in plants abiotic stress resistance by regulating a large number of stress-responsive genes to confer abiotic stress tolerance in plants^70^. The candidate genes mentioned above could be potentially influencing traits of adaptation to highland agro-ecologies in Africa, and the observed selective sweeps might be due to positive natural selection or deliberate selection during the development of inbred lines or source populations.

Multiple candidate genes of known function have been identified on chromosome 6, which include (a) elongation factors that are highly correlated with total lysine content of the endosperm^71,72^; (b) glutathione transferases that catalyze the conjugation of glutathione to xenobiotic compounds in the detoxification process^73^; (c) *G2*-like transcription factors that play a central role in regulating chloroplast development, which contain the green pigment chlorophyll and are responsible for the light-powered reactions of photosynthesis (Liu *et al*. 2016); and (d) basic leucine zipper (*bZIP*) gene family that play important roles in multiple biological processes, such as light signaling, seed maturation, flower development as well as abiotic and biotic stress responses^74^. The four selective sweep regions identified on chromosome 7 consisted of multiple candidate genes, including Kinesin-related proteins (*KRPs*) that play central roles in the transport of various vesicles and organelles in eukaryotic cells^75^; gibberellin 2-oxidases (*GA2oxs*) that regulates plant growth by inactivating endogenous bioactive Gibberellins^76^; the maternally expressed gene (*Meg*) family, which encodes cysteine-rich proteins (*CRPs*)^77^ that are involved in both cell-signaling and antimicrobial processes^78,79^; the Endosperm5 (o5) showed moderate correlation (R^2^ = 0.66) with *Opaque* *2* (*o2*) and affect different aspects of storage protein synthesis in maize^80^; the cellulose synthase (*CesA*) gene family that are primary determinant of wall formation, stalk strength and improve harvest index^81^; carbonic anhydrase (*CA*) that catalyzes the reversible hydration of CO2 into bicarbonate^82^, and implicated in photosynthesis^83^, stomatal conductance and guard cell movement in C3 plants^84^, and providing bicarbonate to the initial carboxylating enzyme phosphoenolpyruvate carboxylase in C4 plants^85^; the basic helix-loop-helix (*bHLH*) transcription factors that play key roles in diverse biological processes, including seed germination, shade avoidance response, flowering time regulation, stress responses and anthocyanins synthesis^86–88^; pentatricopeptide repeat (*PPR*) proteins that have been implicated in RNA editing, RNA processing, translation, photosynthesis, respiration and kernel development^89,90^; the maize D-cyclin gene asceapen1 (asc1) that plays a role in leaf and shoot development^91^ and regulates progression through the G1 phase of the cell cycle^92^.

On chromosome 8, the selective sweeps consisted of several *AP2/EREBP* (*APETALA2/ethylene* responsive element-binding protein) transcription factors that are involved in many different pathways, including drought and high salt concentration^93^, low temperature^94^, diseases^95,96^ and the control of flowering^97^; receptor for activated C kinase that plays a role in plant response to fungal phytopathogens^98^, affect different signal transduction pathways and multiple developmental processes in plants^99^; *MYB* transcription factors that are involved in controlling responses to biotic and abiotic stresses, development, differentiation, metabolism, hormone signal transduction^100,101^; and AP kinase kinase kinase 18 (*MAPKKK18*) that controls plant growth by adjusting the timing of senescence via its protein kinase activity^102^.

## Conclusions

Most of the 298 inbred lines adapted to the African highland ecology showed high level of genetic heterogeneity than expected for lines extracted from S_4_ or later generations, which suggests the need for revising the line development strategy, including line finishing and use of genetically pure parental lines for line development (as compared to landraces, composites and pools that were used in the past); generating reference genotype data as one of the requirements for releasing lines; implementing quality assurance (QA) and quality control (QC) genotyping methods to regularly check genetic purity of key inbred lines during line maintenance; and more frequent use of DH technology in developing breeding lines. The germplasm used in the current study showed clear population structure, primarily by pedigree information and breeding history, and less so by heterotic groups and germplasm type. There was a high level of genetic difference among most pairs of inbred lines although they have a large proportion of alleles in common, which is expected when a limited number of parental lines are used for line development. We identified 944 SNPs that fell within 22 selective sweep regions, which harbored 265 annotated genes whose functions provide clues on the adaptation of the tropical maize to the African highlands. Molecular diversity indices computed across multiple categorical variables using SNPs that fell within the selective sweeps showed a two-fold reduction on polymorphic sites and nucleotide diversity in two of the three groups predicted based on the model-based STRUCTURE as compared to the genome-wide SNPs. Such thorough analyses on the genotypic data depict a significant contribution of this study to the available knowledge on selective sweeps in maize. Results from this study provide valuable information for further improvement of highland maize breeding programs in Africa, including the need for revising the line development strategy, diversifying parental lines for developing new inbred lines, and verifying genetic purity of newly fixed inbred lines using QC genotyping.

## Supplementary information

Supplementary Information

Supplementary Information

Supplementary Information

Supplementary Information

Supplementary Information

Supplementary Information

Supplementary Information

## Data Availability

All relevant data are within the paper and its Supporting Information Files.
