# Supplementary material for: Molecular diversity and selective sweeps in maize inbred lines adapted to African highlands
Source: Sci Rep. 2019 Sep 17;9:13490. doi: 10.1038/s41598-019-49861-z (PMC6748982; doi:10.1038/s41598-019-49861-z)

Molecular diversity and selective sweeps in maize inbred lines adapted to African highlands

Dagne Wegary, Adefris Teklewold Chere, Boddupalli M. Prasanna, Berhanu T. Ertiro, Nikolaos Alachiotis, Demewez Negera, Geremew Awas, Demissew Abakemal, Veronica Ogugo, Manje Gowda, and Kassa Semagn

Supplementary Figure S1. Summary of the proportion of heterogeneity and genetic purity in 298 inbred lines, estimated using 235,019 polymorphic SNPs in Dataset-2.

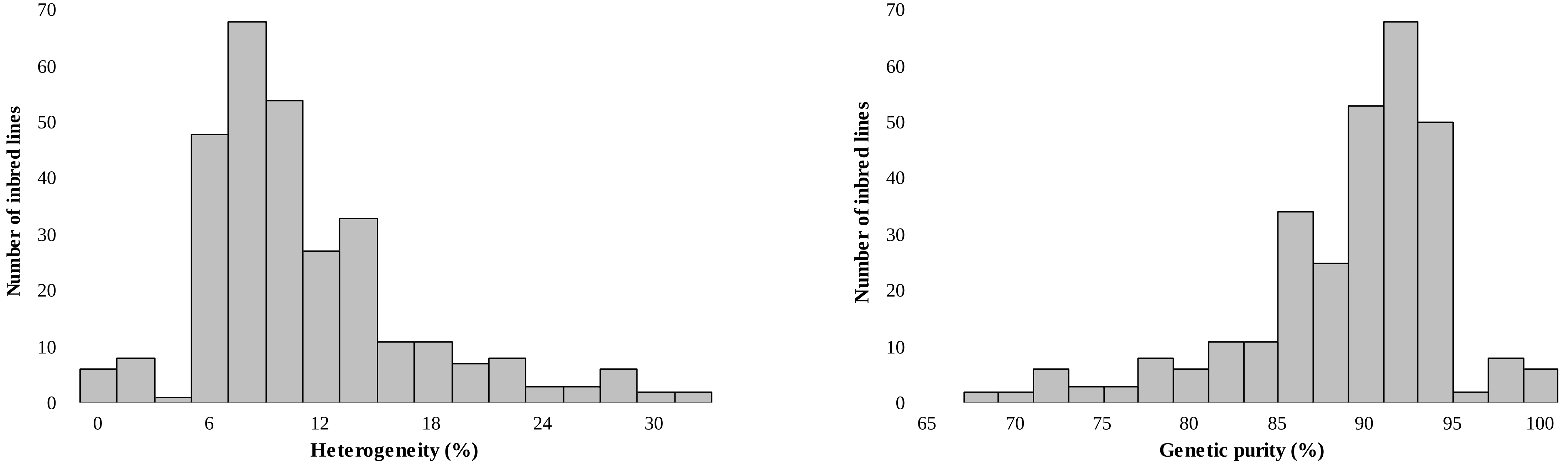

Supplementary Figure S2. Neighbor-joining tree of 298 inbred lines based on identity-by-state (IBS) genetic distance matrix computed from 235,019 polymorphic SNPs, each with minor allele frequency >0.05.

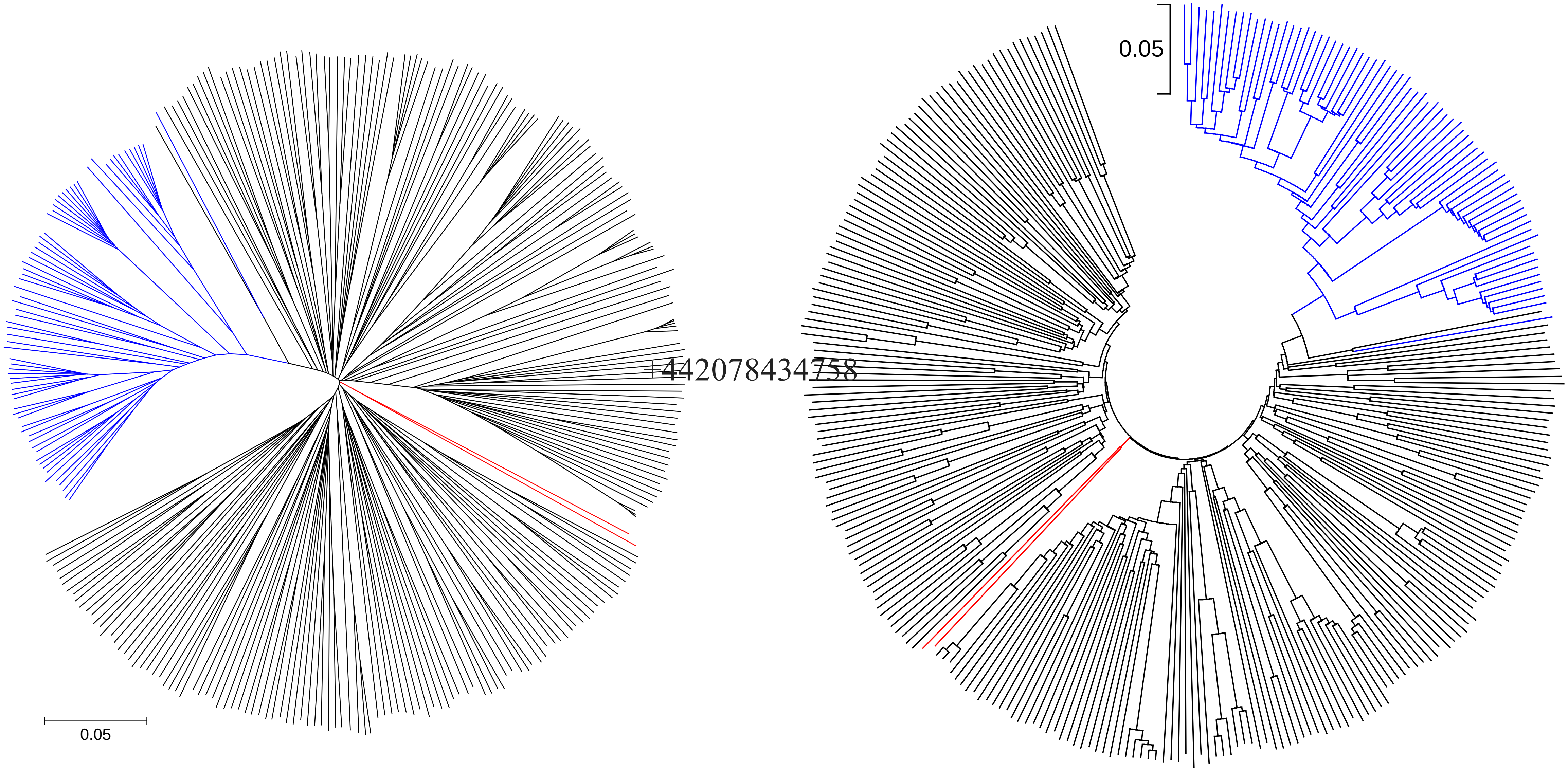

a) NJ tree for 298 lines based on group membership at K=2 (G1 = black; G2 = blue; ungrouped = red)

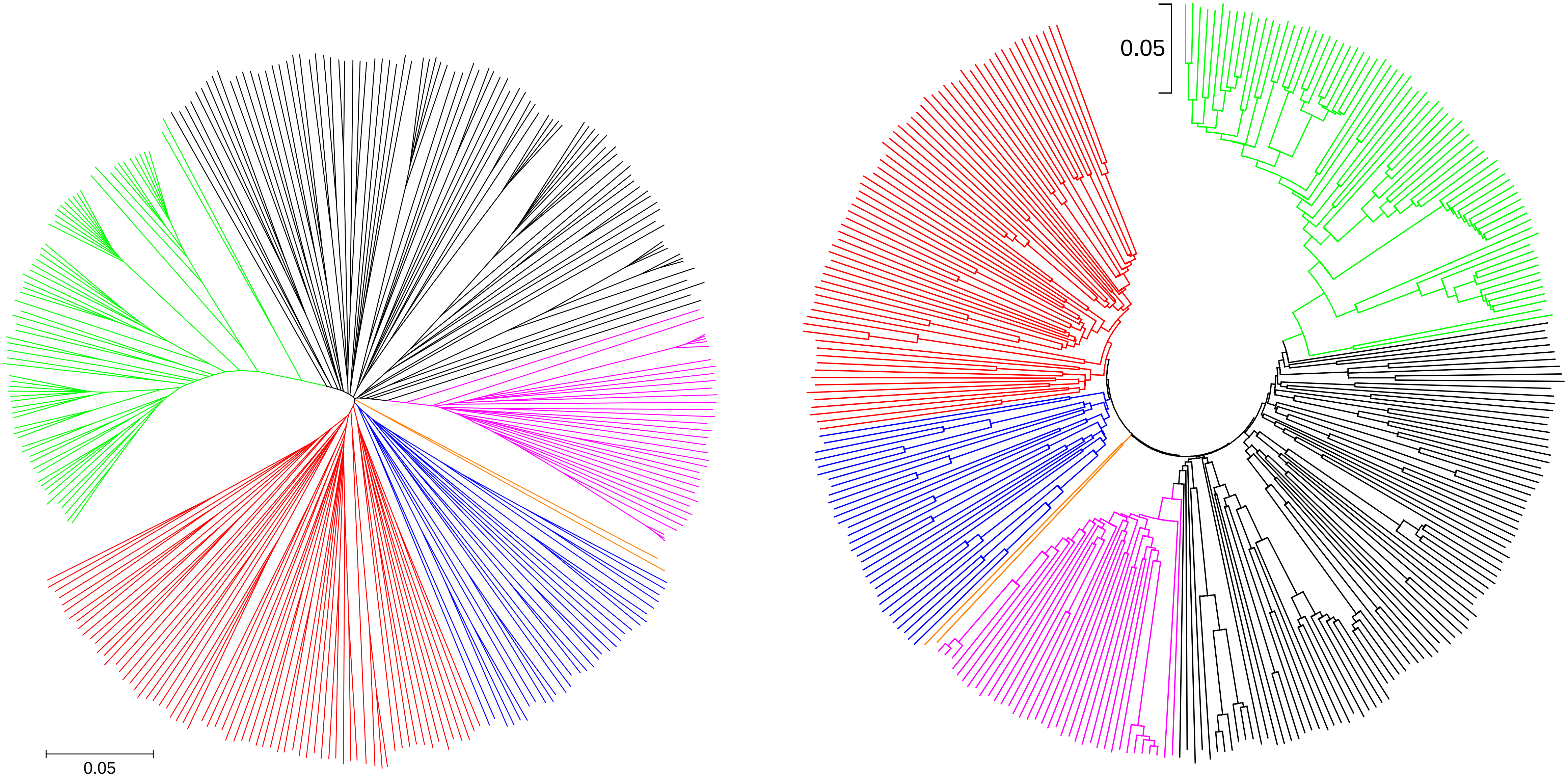

(b) NJ tree for 298 lines based on NJ cluster analysis (G1-A = black; G1-B = red; G1-C = blue; G2 = green; G3 = pink or fuchsia; Ungrouped = orange). **Note G1, G2 and G3 are the three groups at K=3**

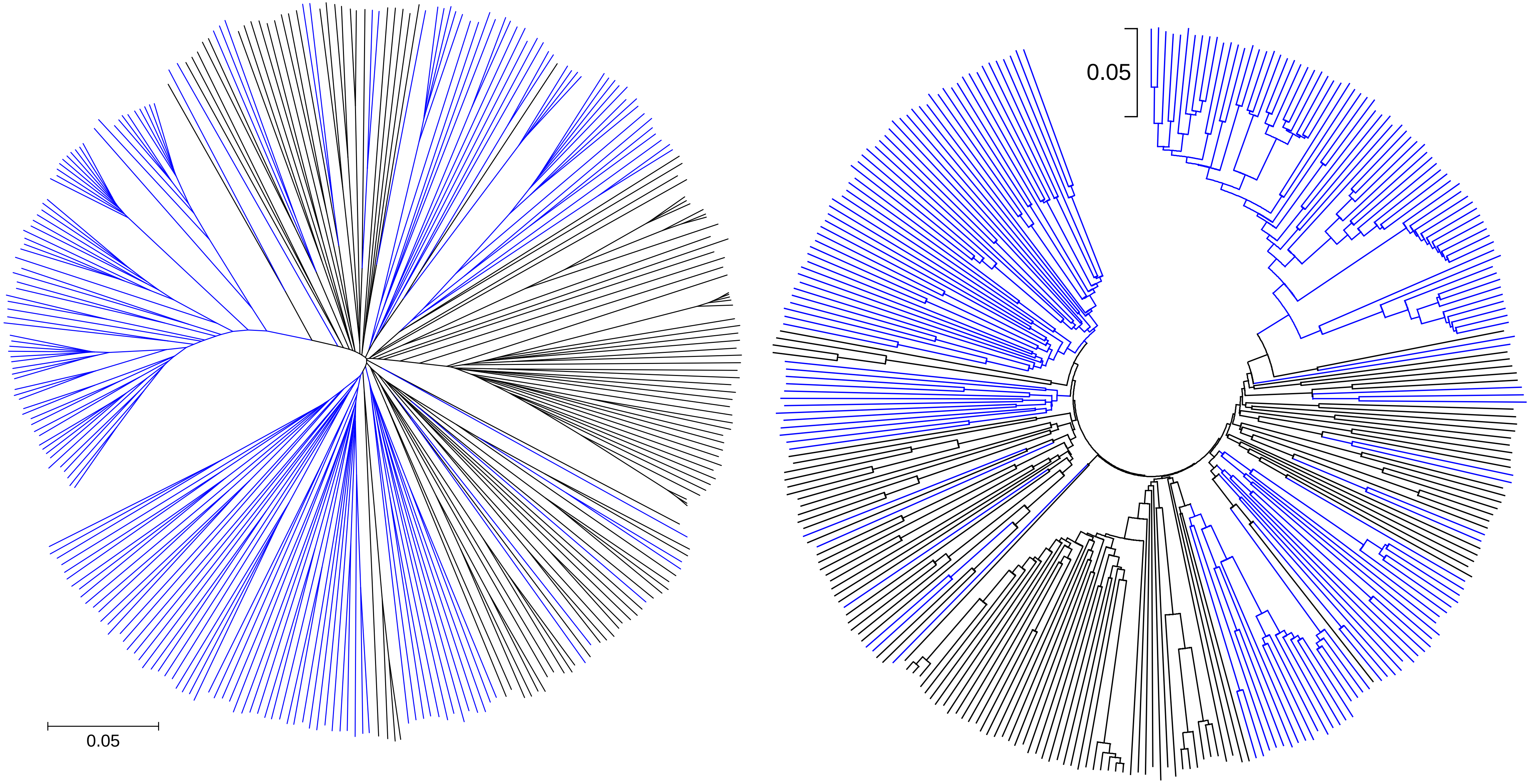

(c) NJ tree for 298 lines based on germplasm type (Non-QPM= black; QPM = blue)

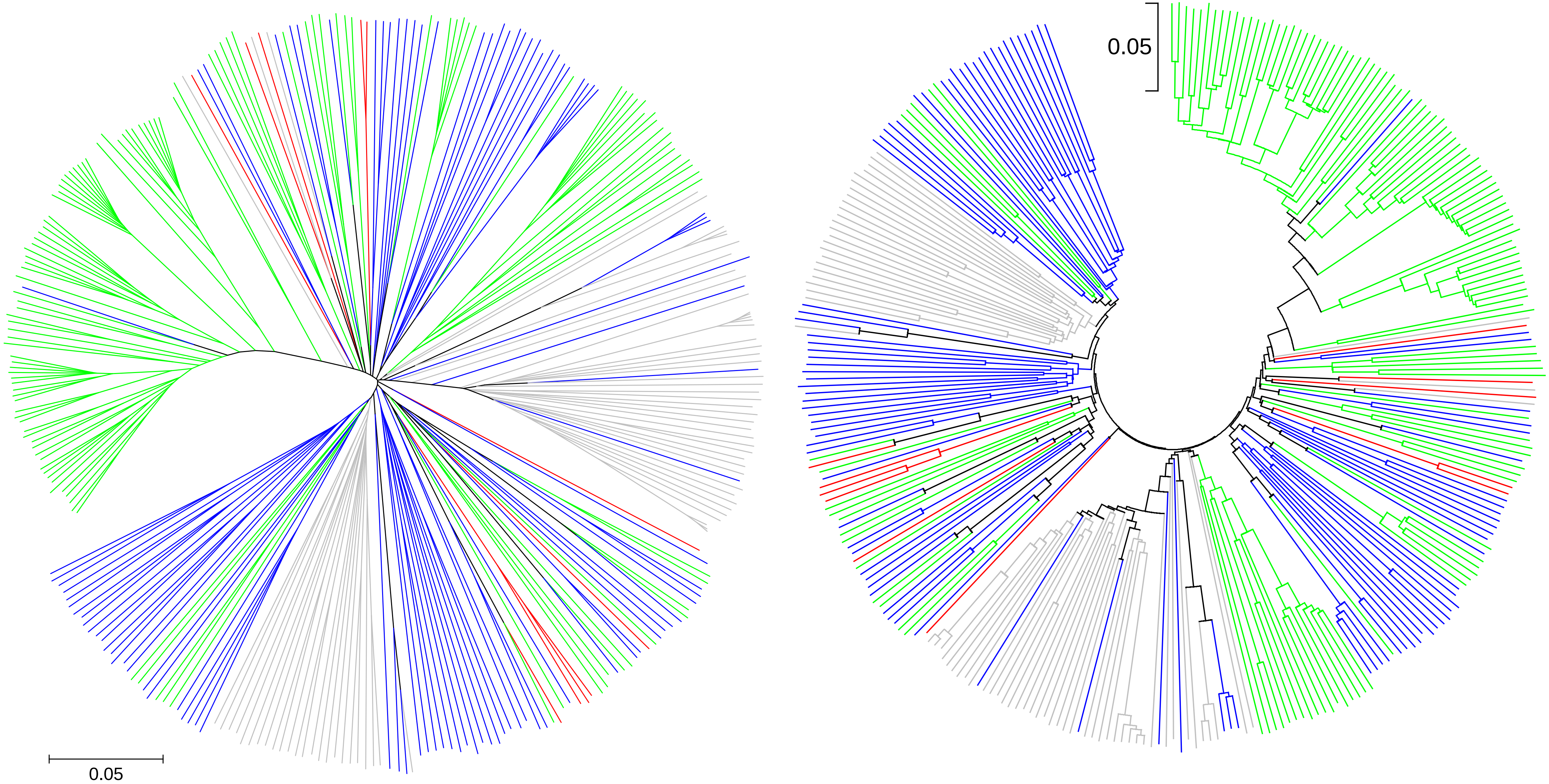

(d) NJ tree for 298 lines based on heterotic grouping (Heterotic group A = green; B = blue; AB = red; Unknown: silver)

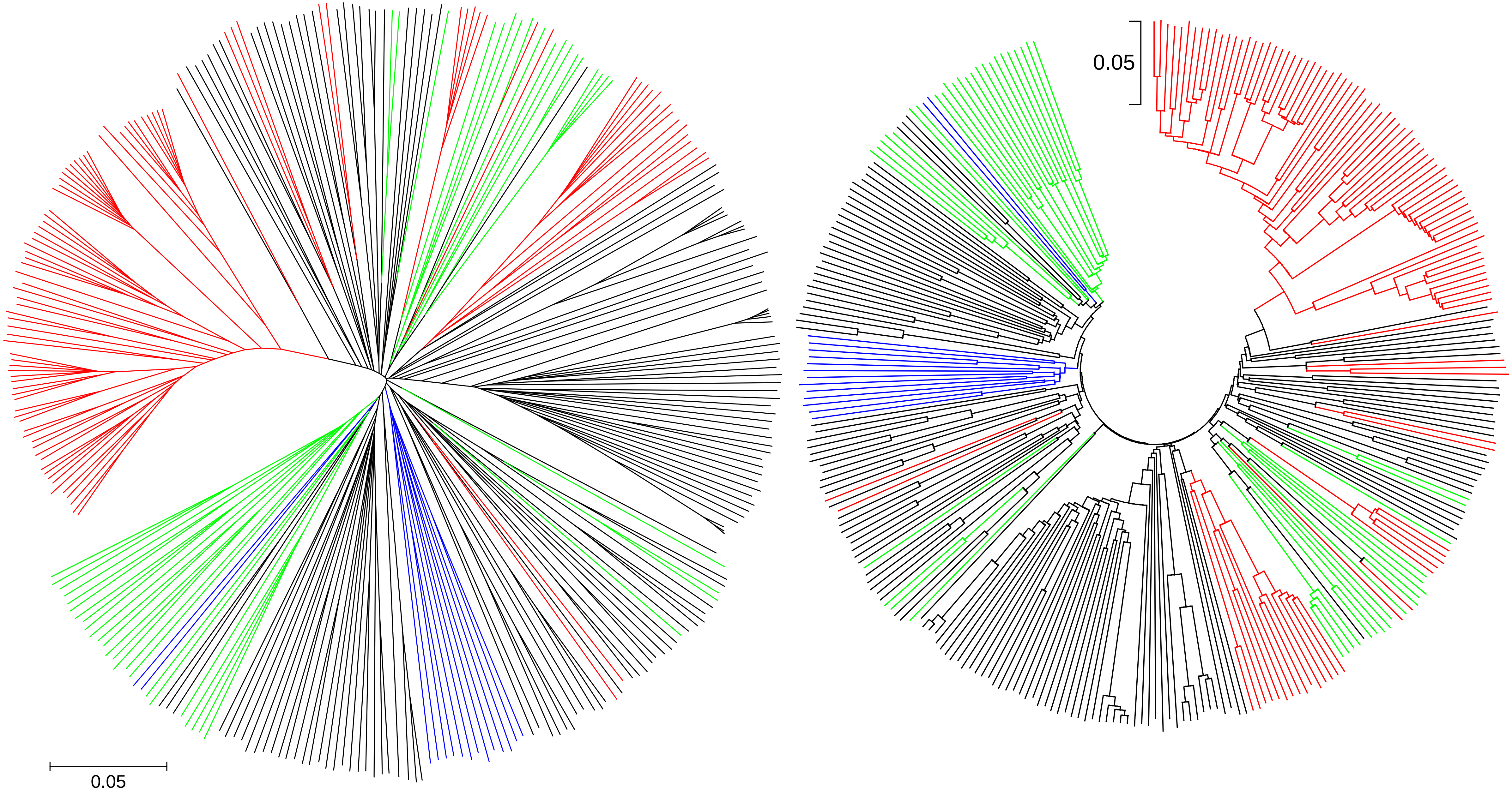

(e) NJ tree for 298 lines based on genetic background of QPM donor parents: non-CML (black); CML144 (red); CML159 (blue) and CML176 (green).

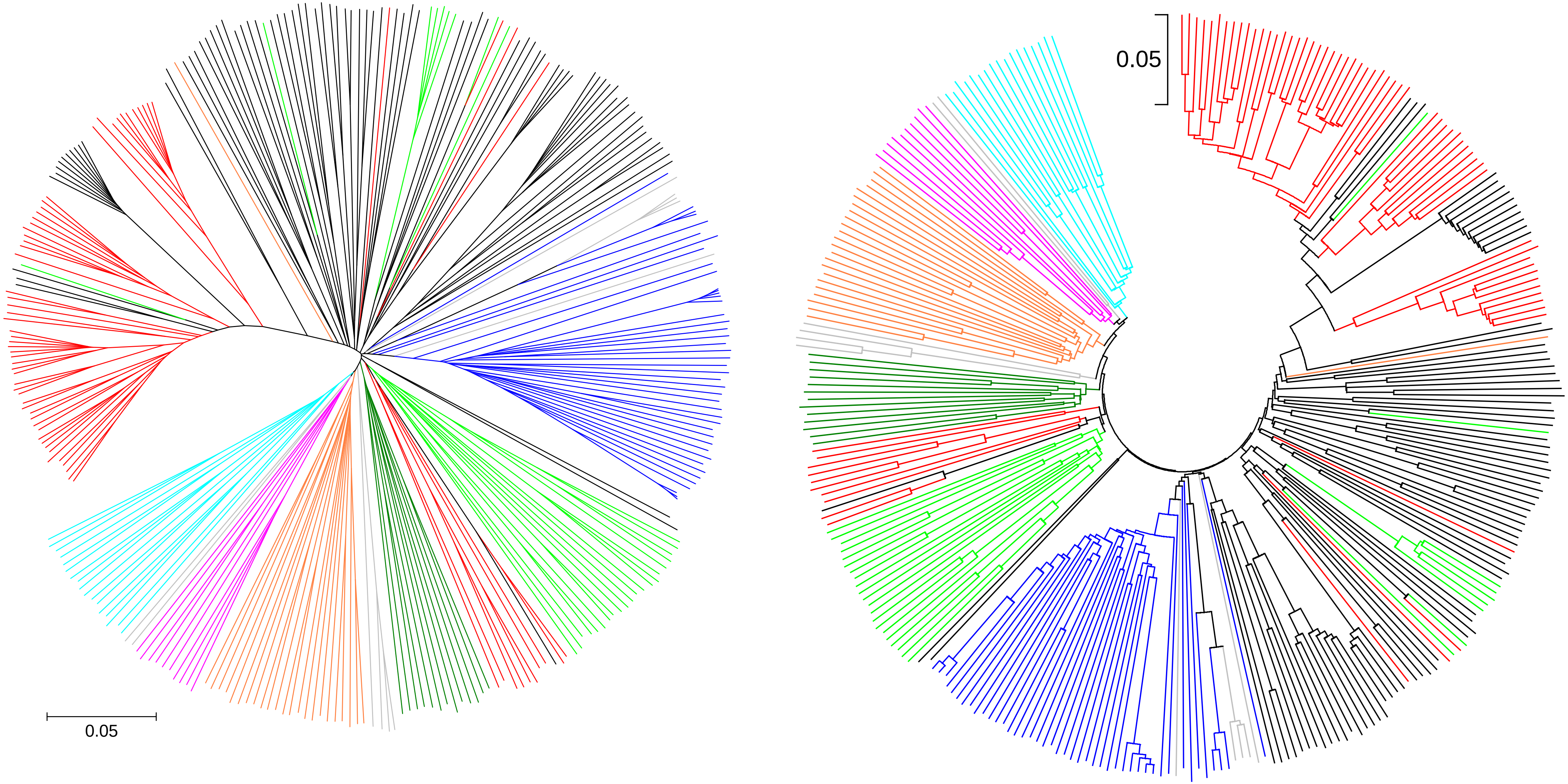

(f) NJ tree for 298 lines based populations (genetic backgrounds of the recurrent parents). Ecuador-573 (red); Kitale-SYN (light green); Pool-9A (blue); Pool-9A-SR (black); Pop-502-SR (aqua); SADVLA (pink); SUSUMA (orange); Tuxpeno (dark green).

Supplementary Figure S3. Plot of PC_1 (11.3%) and PC_2 (5.4%) from a principal component analysis of 298 inbred lines using 235,019 polymorphic SNPs, each with minor allele frequency >0.05. The plots were made using the following categorical variables: (a) the model-based population STRUCTURE at K=2; (b) NJ cluster analysis; (c) type of germplasm; (d) heterotic grouping; (e) genetic background of the QPM donor parents; and (f) genetic background of the recurrent parents. See Supplementary Table S1 for details of each group membership.

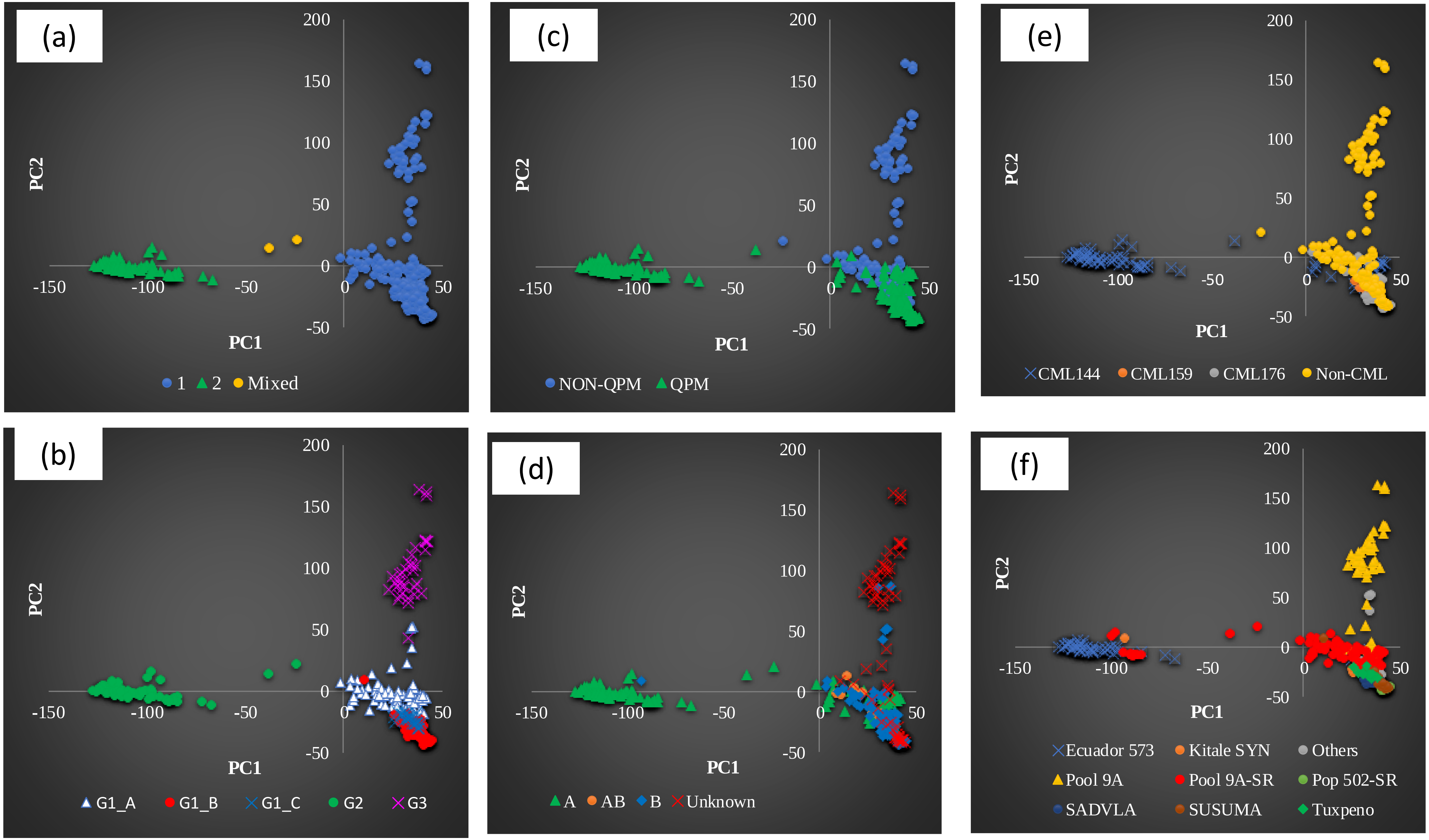

Supplement: Supplementary file 1 — Supplementary Information [file 41598_2019_49861_MOESM1_ESM.pdf]
